# Patterns of chromosomal copy-number alterations in intrahepatic cholangiocarcinoma

**DOI:** 10.1186/s12885-015-1111-6

**Published:** 2015-03-14

**Authors:** Cyril Dalmasso, Wassila Carpentier, Catherine Guettier, Sophie Camilleri-Broët, Wyllians Vendramini Borelli, Cedália Rosane Campos dos Santos, Denis Castaing, Jean-Charles Duclos-Vallée, Philippe Broët

**Affiliations:** 1 Laboratoire de Mathématiques et Modélisation d’Evry (LaMME), Université d’Evry Val d’Essonne, UMR CNRS 8071, USC INRA, Evry France; 2 Plate-forme Post-Génomique P3S, UPMC, Faculté de Médecine, Paris, France; 3 DHU Hepatinov, Centre Hépato-Biliaire, Hôpital Paul Brousse, AP-HP, Villejuif, France; 4 Faculté de Médecine, Univ. Paris-Sud, Kremlin-Bicêtre, France; 5 Department of Pathology, McGill University, Montreal, Canada; 6 Faculdade de Medicina, Hospital São Lucas da Pontifícia Universidade Católica do Rio Grande do Sul, Porto Alegre, Brazil; 7 DHU Hepatinov, UF Biostatistiques, Hôpital Paul Brousse, AP-HP, Villejuif, France; 8 INSERM UMR-669, Villejuif, France

**Keywords:** Cholangiocarcinoma, DNA copy-number, Genomic

## Abstract

**Background:**

Intrahepatic cholangiocarcinomas (ICC) are relatively rare malignant tumors associated with a poor prognosis. Recent studies using genome-wide sequencing technologies have mainly focused on identifying new driver mutations. There is nevertheless a need to investigate the spectrum of copy number aberrations in order to identify potential target genes in the altered chromosomal regions. The aim of this study was to characterize the patterns of chromosomal copy-number alterations (CNAs) in ICC.

**Methods:**

53 patients having ICC with frozen material were selected. In 47 cases, DNA hybridization has been performed on a genomewide SNP array. A procedure with a segmentation step and a calling step classified genomic regions into copy-number aberration states. We identified the exclusively amplified and deleted recurrent genomic areas. These areas are those showing the highest estimated propensity level for copy loss (*resp.* copy gain) together with the lowest level for copy gain (*resp.* copy loss). We investigated ICC clustering. We analyzed the relationships between CNAs and clinico-pathological characteristics.

**Results:**

The overall genomic profile of ICC showed many alterations with higher rates for the deletions. Exclusively deleted genomic areas were 1p, 3p and 14q. The main exclusively amplified genomic areas were 1q, 7p, 7q and 8q. Based on the exclusively deleted/amplified genomic areas, a clustering analysis identified three tumors groups: the first group characterized by copy loss of 1p and copy gain of 7p, the second group characterized by 1p and 3p copy losses without 7p copy gain, the last group characterized mainly by very few CNAs. From univariate analyses, the number of tumors, the size of the largest tumor and the stage were significantly associated with shorter time recurrence. We found no relationship between the number of altered cytobands or tumor groups and time to recurrence.

**Conclusion:**

This study describes the spectrum of chromosomal aberrations across the whole genome. Some of the recurrent exclusive CNAs harbor candidate target genes. Despite the absence of correlation between CNAs and clinico-pathological characteristics, the co-occurence of 7p gain and 1p loss in a subgroup of patients may suggest a differential activation of EGFR and its downstream pathways, which may have a potential effect on targeted therapies.

## Background

Intrahepatic cholangiocarcinomas (ICC) are malignant tumors arising from intra-hepatic bile duct epitheliums, either from large or small bile ducts. ICC is a relatively rare malignant tumor but represents around 10% of the primary hepatic malignancies worldwide [1]. There is a dramatic geographic disparity for the incidence rates of ICC that mainly correlates with difference in environmental risk factors. The highest incidence is observed for Asian countries in areas with liver fluke endemic infection. In western countries, the age-adjusted incidence and mortality of ICC have increased over the last decades [2-5] and classical risk factors reported in the literature are chronic biliary tract disorders such as primary sclerosing cholangitis, biliary-duct cysts, hepatolithiasis. Others non-biliary related conditions have also been discussed [1,6]. A recent meta-analysis reported [5] that cirrhosis, chronic hepatitis B and C, alcohol use, diabetes and obesity are risk factors for ICC. However, for the majority of the patients, no risk factor can be identified.

For most of the patients, the disease remains asymptomatic or paucisymptomatic for a long period of time and is diagnosed at advanced stages which leads to a poor prognosis. Surgical resection or liver transplantation represent the only curative options that can be offered to a fraction of the patients. In some locally advanced cases, surgery may be proposed after primary systemic chemotherapy or portal vein embolization. After curative resection, there is a high rate of relapse that favors the use of adjuvant therapies. Nevertheless, the wide patient-to-patient variability in tumor characteristics and outcomes raises a challenge for refining the use of innovative adjuvant therapy.

Recent advance in genomic science has offered new opportunities for deciphering cancer diseases and identifying molecular prognostic or predictive markers. In practice, these genomic technologies provide a means of finding novel actionable targets and defining new therapeutic strategies based on molecular classifications. For several solid tumors, such as lung and colon carcinomas, the use of targeted therapy has leaded to major changes in the management of patients. Despite major recent genomic studies, the molecular portrait of ICC remains incomplete with few actionable genomic abnormalities but some new early phase clinical trials with targeted therapies are currently underway [7,8].

Before the widespread use of high throughput genomic technologies, published studies on cholangiocarcinomas have focused on a limited number of genetic alterations and reported mutations in KRAS, TP53, SMAD4, CDKN2A, CDH1 or BRAF genes [9-13]. High level amplifications of HER2 and CyclinD1 and overexpression of MET have been also described in few cases [14-17]. However, the series were often heterogeneous (mixing intra and extra-hepatic cases) and of small sample size which may explain the wide variations in prevalence reported for these alterations.

In the recent years, a tremendous effort has been made to investigate molecular alterations of ICC using genome-wide technologies. Among them, two gene expression-based classifications have been published [18,19]. From a series of 104 surgically resected cholangiocarcinomas (not restricted to intrahepatic cases), Anderson *et al.* [18] proposed a gene expression signature that identified a high risk group of patients. The most malignant phenotype was characterized by up-regulation of the tyrosine kinase signaling pathways (HER2, EGFR, MET). In another study, Sia *et al.* [19] analyzed a series of 149 archived formalin-fixed tumor tissues and reported the presence of two distinct transcriptional classes: the so-called *inflammation class* characterized by activation of inflammatory signaling pathways and the so-called *proliferation class* characterized by activation of oncogenic signaling pathways (including EGFR and MET), the latter being associated with a worse outcome.

From a genome-centered perspective, recent series aimed to find new so-called driver mutations using either targeted or whole-genome/exome sequencing technologies. Differences regarding the sequencing strategies and the tissue preservation methods (frozen or formalin-fixed paraffin-embedded material) may explain some discrepancies between the reported frequencies of driver mutations. The most frequent mutations are found in either classical tumor-associated genes (KRAS, TP53, PTEN, BRAF) or newly reported genes such as: IDH1/2, BAP1, ARID1A, ERRFI1, PBRM1 [20-29]. Fusions have also been demonstrated for FGFR2 gene [21,30]. In a study comparing fluke and non-fluke associated cholangiocarcinomas [28], Chan-on *et al.* reported a higher frequency of BAP1 and IDH1/2 in non-fluke associated cases. In these latter group, the authors showed the existence of a lower overall mutation burden. This finding raised the question of different mechanisms of biliary carcinogenesis. In the same study, Chan-on *et al.* also investigated copy number alterations (CNAs) from a small subset of 15 non-O. Viverrini cholangiocarcinomas and observed multiple alterations that emphasized the potential role of CNAs in biliary carcinogenesis.

To date, few studies have analyzed whole-genome CNAs in ICC [7,8]. Among them, Ross *et al.* [26] described focal amplifications of MCL1 and MET. Sia *et al.* [19] identified frequent CNAs and reported the poor outcome associated with 14q22.1 deletion. It is worth noting that both series have been performed on paraffin-embedded material. The use of this material has the great advantage of allowing analysis of large retrospective series but leads to underestimate the occurrence of low-level copy-number alterations. This is particularly challenging for this tumor type which is often characterized by the presence of an abundant stroma reaction.

In order to characterize the patterns of chromosomal alterations in ICC, we conducted a genome-wide CNAs study using frozen material in a surgical series of patients with ICC. We evaluated the relationships between clinico-pathological characteristics and copy number changes.

## Methods

### Patients and samples

The study included 53 patients with intrahepatic cholangiocarcinoma who underwent surgical resection with curative intent from 2003 to 2009 at the Centre Hépato-Biliaire (Hepatinov, Villejuif, France). Informed consent was obtained for each patient. Retrospective collection of clinical data was approved by the Comité d’éthique du Groupe Hospitalier Paris-Sud. Patient demographics, symptoms, anamnesis, operative data, tumor pathology and disease recurrence were obtained from hospital medical records. Stage grouping was assessed *a posteriori*, by using the AJCC/UICC TNM (7th edition) [31]. Tumor samples were immediately snap frozen and stored at −80°*C* until retrieved. All frozen samples underwent pathological examination by an experienced pathologist (CG). Samples that contained a minimum of 20% of tumor cells were used in the analyses whereas the remaining cases were used as negative controls.

DNA extraction was performed using PureLink Genomic DNA Mini Kit (Invitrogen, Camarillo, CA, USA) and were hybridized on Infinium HumanExome BeadChip (Illumina Inc., San Diego, CA, USA), according to the manufacturer’s specifications. All experiments were performed at the P3S Pateform UPMC, Paris, France). The exonic content of the HumanExome chip consists of more than 250,000 markers focusing on exonic regions. Only 47 high-quality samples were hybridized. The genome-wide analysis has been performed on 42 samples (≥20*%* of tumor cells).

### Statistical analysis of SNP array data

The arrays were scanned with an iScan system [32]. We used the intensity signals obtained from a total of 242,296 exonic autosomal probes. Normalized log R Ratio (LRR) and B allele frequencies (BAF) were obtained from BeadStudio [32] as described in [33].

#### CNA calling procedure

Various methods have been proposed to study CNAs using SNP arrays but the final calling step still remains a challenging task, especially in tumors with a high stromal content. For this study, we have implemented a tree-based algorithm to classify genomic regions into four meaningful and easily interpretable CNA states: copy loss (Loss), copy neutral (Neutral), copy gain (Gain) and copy neutral loss of heterozygosity (CNLOH or copy neutral allelic loss).

**Figure 1:**
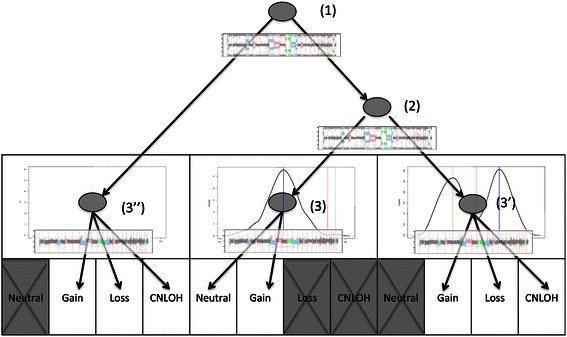
**Flowchart of the calling algorithm used to classify each segment.** Steps (1) and (2) are based on the BAF values while the final calling (steps (3), (3’) and (3")) is based on the LRR values.

The basic two consecutive steps of the procedure are the segmentation step (identification of the breakpoints in the data) and the calling step (allocation of the segments to one of the CNA states). For the segmentation step, the data were processed using a joint segmentation of the bivariate signal (LRR and BAF) [34]. Broadly speaking, a recursive binary segmentation [35] has been used to identify a list of candidate breakpoints and the final segmentation has been obtained from a pruning step using dynamic programming [36]. For the calling step, we considered a tree-based algorithm (summarized in Figure 1) with five decision nodes allowed to assign each segment a CNA state. The BAF values were used at two decision nodes (labeled (1) and (2)) to distinguish segments with a loss of heterozygosity (LOH), segments with an even number of copies for both alleles or segments with an odd number of copies. The LRR values were used at three decision nodes (labeled (3), (3’) and (3”)) to classify segments as copy loss, copy neutral or copy gain. The decision nodes are described in more details below.

To identify heterozygous SNPs, a kernel estimate of the BAF density has been considered. The two extreme values (i.e. the closest to zero and one) corresponding to local minima, were used as thresholds to exclude homozygous SNPs. For each segment (decision node labeled (1) in Figure 1), a binomial test was performed to identify loss of heterozygosity (segments for which the proportion of heterozygous SNPs was significantly lower than the proportion estimated for the whole genome). For segments without LOH, it was expected that even (*resp.* odd) copy number values were giving rise to unimodal (*resp.* bimodal) BAF distribution. Thus, in order to identify these two types of segments, a combination of the Hartigan’s dip test of unimodality [37] and the Wilcoxon test was performed. In practice, if one of the two tests was significant, the segment was considered as having odd copy number values (decision node labeled (2) in Figure 1).

Segments with even copy number values can be classified as either copy neutral or copy gain (with at least two additional copies). To detect these copy gains, LRR medians were computed for all segments having even copy number values. The copy gains, giving rise to extreme values, were detected by a Grubbs’ test for outliers (decision node labeled (3) in Figure 1). In contrast, segments having odd copy number values can be classified as copy loss, copy gain or CNLOH (either mixture of normal cells or tumoral heterogeneity). Based on the LRR values corresponding to copy neutral segments identified previously (at decision node labeled (3)), an equivalence test was performed to identify CNLOH segments. Then, the remaining segments were classified as copy gain (*resp.* loss) if the LRR median was greater (*resp.* lower) than the copy neutral LRR median (decision node labeled (3’) in Figure 1). Finally, to distinguish CNLOH from LOH with copy number changes, one-sided Wilcoxon tests were performed for each segment with loss of heterozygosity (decision node labeled (3”) in Figure 1).

To take into account the multiple testing problem resulting from the large number of statistical tests that are performed for the whole procedure, we considered a weighted Bonferroni procedure. The adjusted level used for each segment is defined as $\frac {\sum {n_{i}}}{n_{i}}\alpha $ (where *n*_*i*_ is the segment size). Then, these adjusted levels are divided by the maximum number of statistical test that may be performed for each segment. The family wise error rate for the whole procedure is then controlled at an *α* level of 5%.

For the clustering and association analyses and in order to summarize genomic information while keeping a sufficient level of resolution, copy number states was inferred from applying the majority voting procedure based on their cytoband location according to the Human NCBI Genome Build 37.1 In total, 768 cytobands were analyzed.

#### Chromosomal cytoband aberration patterns

The aim of this analysis was to identify patterns of recurrent CNAs considering jointly the chromosomal propensity for deletion and amplification. More precisely, our interest was to select recurrent so-called *exclusively* deleted (*resp.* amplified) cytobands that were those with the highest level for copy loss (*resp.* copy gain) together with the lowest level for copy gain (*resp.* copy loss). This choice relied on the hypothesis that these *exclusive* behaviors reflect a selective advantage for tumor growth for one state (*e.g.* copy loss) associated with a selective disadvantage of the converse state (*e.g.* copy gain).

In order to represent the cytoband’s propensity for the occurrence of either copy loss or copy gain across all the ICC tumor samples, we considered a multi-class model-based approach that describes the joint propensity of a given cytoband for being deleted/unmodified/amplified [38]. Broadly speaking, the latent model describes nine classes (3×3) that corresponds to three levels (low/medium/high) of copy loss and copy gain. The allocation of a cytoband to one of the nine classes was performed using the Bayes classification rule that assigned each cytoband to the class to which it had the highest probability of belonging. The final results of this analysis is a set of *exclusively* deleted and amplified cytobands.

In order to determine if these *exclusively* deleted and amplified cytobands were able to identify clusters of tumor samples, we considered a model selection procedure which infers the unknown number of tumor clusters using the most relevant subset of *exclusively* deleted and amplified cytobands [39]. We selected the model that gave the optimal number of tumor samples together with the optimal set of relevant cytobands according to the Bayesian information criterion.

#### General statistical methodology

In order to increase the statistical power of the association analysis between CNAs and clinico-biological parameters, the few copy losses of a cytoband considered as *exclusively* amplified were gathered with those having a modal status. The converse was also done for a cytoband considered as *exclusively* deleted. For each cytoband, the relationships between clinico-pathologic features and CNAs were assessed using either the Pearson’s chi-squared test with Yates’ correction for continuity (categorical variables) or the analysis of variance (continuous variables with log-transformation). The null hypothesis is that the CNA distribution is the same for all the modalities (or levels) of the variable of interest.

Relapse-free survival (RFS) was calculated from the date of diagnosis until date of first relapse or last follow-up examination. All other events were censored. Kaplan-Meier analysis was carried out to generate RFS curves. Univariate proportional hazard model (Cox model) analysis was performed to assess the prognostic influence of clinical and biological variables. For the analysis of the set of cytobands and to address the multiple testing problem, we controlled the family wise error rate at an *α* level of 5%. Analyses were performed with R 2.15.3 software [40].

## Results and discussion

### Clinical characteristics

The median age at diagnostic was 60 years (range: 27-81). 28 patients were men. Among the 53 patients, the following potential etiological factors were obtained from patients’ anamnesis: hemochromatosis (two patients), primary sclerosing cholangitis (one patient), hepatitis HCV-related liver cirrhosis (one patient), Wilson disease-related cirrhosis (one patient). Additionally, pathological examination of the non tumoral liver tissue has found cirrhosis in three patients, sclerosing cholangitis histological lesions in two patients. One patient, having a history of HBV infection, was diagnosed with a biliary cystadenocarcinoma arising in a cystadenoma. No patient was known to have had prior exposure to liver flukes. Most of our patients (42/53) did not show any pre-existing liver disease, concordant with the literature [41].

Symptoms of biliary obstruction were observed in eight cases at the time of diagnosis. Surgery was performed after portal vein embolization in 12 cases and after primary chemotherapy in 20 cases. From the latter, significant regression fibrosis was observed in three cases on histological examination. We decided then to analyze for genomic analyses the whole series as a homogeneous group. The surgical treatment was segmentectomy (5 patients), transplantation (3 patients) and hemihepatectomy or extended hemihepatectomy for the remaining patients. Lymph node evaluation has been performed in 33 cases.

From macroscopic examination, the median specimen weight was of 670 grams (range: 78-2,653). The median tumor size was 8.0 cm (range: 2.3-25.0). Twenty-seven patients had multiple tumors, including 20 patients with more than three nodules. Eleven patients had macrovascular invasion. Macroscopic intraductal growth was observed in eight specimens. Thirty-two patients had surgical margin infiltration. Histology showed well differentiated adenocarcinomas in 16 cases.

With the exception of five papillary carcinomas and two sarcomatoid carcinomas, most of the samples were diagnosed as tubular adenocarcinomas. Histological vascular invasion and perineural infiltration were observed in 29 over 48 patients and 11 over 43 patients, respectively. From the medical records, the stage has been evaluated according to the AJCC/UICC 7th edition for 52 patients: stage I (6/52, 11%), stage II (31, 60%), stage III or IV (15, 29%).

The median time to recurrence was 19 months. The relapse-free survival rate was 46.2% (95% confidence interval: 33.0%-64.7%) at 24 months.

### Calling procedure

**Figure 2:**
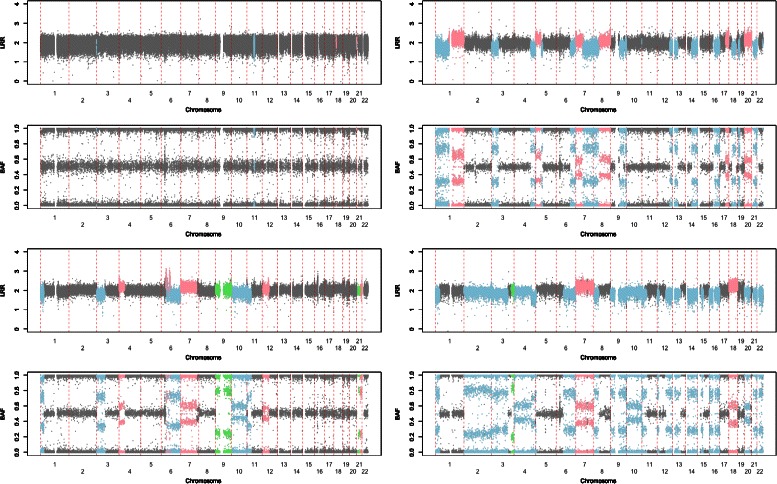
**LRR values and BAF values all along the 22 autosomes for four samples with different proportions of tumoral cells: less than 20% (top left panel), 30% (top right panel), 40% (bottom left panel) and 60% (bottom right panel).** Copy neutral, copy gain, copy loss and CNLOH are in gray, pink, blue and green, respectively.

In five cases with no or very few tumor cells, we observed no CNA which reflects the high specificity of our procedure. We tested the relationship between CNAs and the percentage of tumor cells distributions across the tumor samples. There was no significant relationship (p=0.40) between the fraction of the genome altered (copy loss or gain) and the percentage of tumor cells which reflects the good behavior of our classification procedure for the chosen cut-off (20% of tumor cells). Figure 2 displays BAF and LRR values together with the allocation states for four samples with different proportions of tumoral cells contents: less than 20% (top left panel), 30% (top right panel), 40% (bottom left panel) and 60% (bottom right panel). Copy neutral, copy gain, copy loss and CNLOH are in gray, pink, blue and green, respectively. From this figure, we can see the interest of considering simultaneously BAF and LRR values for the calling procedure. Out of the 42 cases analyzed, 15 tumors showed some copy-neutral events with very few recurrent event (less than three).

### Landscape of copy number alterations

**Figure 3:**
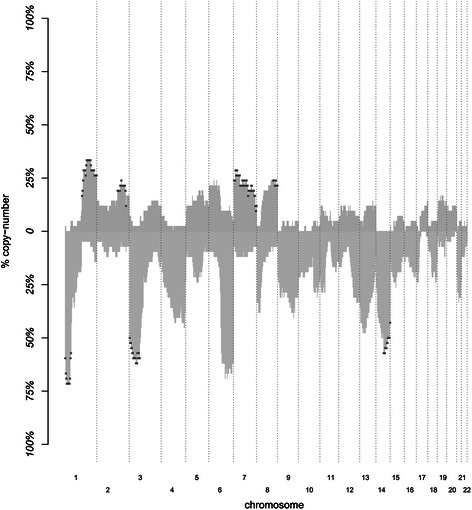
**Frequencies of chromosomal aberrations.** The frequencies of amplification (light pink) and deletion (light blue) over the ICC samples are plotted and ordered, according to the chromosomal order (x-axis) from 1 pter to 22 qter. Exclusively deleted and amplified cytobands are underlined in purple.

In the following, we report the pattern of cytoband aberrations across the entire genome. The median rate of cytoband CNAs per patient was of 27.4% (range 0%-70.8%). Figure 3 displays the frequencies of copy losses and copy gains across the whole genome from 1pter to 22qter. From visual inspection, the frequency rates of deleted cytobands were higher than those of amplified cytobands. The global CNAs portrait is broadly concordant with previous series [19,42].

Results of the chromosomal cytoband pattern analysis are shown in Table 1 that displays, for the nine classes, the joint estimated average probabilities for copy loss, neutral and copy gain. Probability for copy gain ranges from 0.9% to 26.8% whereas for copy loss it ranges from 10.2% to 69.3%. Applying the Bayes classification rule, 42 (5.9%) genomic cytobands were classified as *exclusively* deleted and 98 (12.8%) as *exclusively* amplified. An *exclusively* deleted cytoband has the highest frequency for copy loss (69.3%) together with the lowest frequency for copy gain (0.9%). An *exclusively* amplified cytoband has the highest frequency for copy gain (26.8%) together with the lowest frequency for copy loss (10.2%).

**Table 1:** **Joint estimated probabilities of copy loss (L)/copy neutral (N)/copy gain (G) for the 9 classes**

	*Low*	*Medium*	*High*
*Low*	L:13.6%, N:83.9%,	L:35.0%, N:63.1%,	**L:69.3%, N:29.8%,**
	G:2.5%	G:1.9%	**G:0.9%**
*Medium*	L:12.1%, N:75.2%,	L:32.2%, N:58.0%,	L:66.5%, N:28.6%,
	G:12.7%	G:9.8%	G:4.9%
*High*	**L:10.2%, N:63.0%,**	L:28.0%, N:50.5%,	L:62.0%, N:26.6%,
	**G:26.8%**	G:21.5%	G:11.4%

The three percentages given in each cell represent the frequency of copy loss/neutral/gain, respectively. The results for the *exclusively* deleted and amplified classes are in bold.

In the following, we will focus on genes located on either *exclusively* deleted or *exclusively* amplified cytobands. These *exclusively* deleted and amplified cytobands are reported in Table 2 with their corresponding mean percentage of copy loss and copy gain over the genomic area. The *exclusively* deleted cytobands were observed on 1p36.33-1p35.1, 3p26.3-3p14.25 and 14q24.1-14q32.33. It is worth noting that the 6q deleted area was not considered as *exclusively* deleted since this genomic area showed a medium level of amplification. The *exclusively* amplified cytobands were observed on 1p11.2-1p41.1, 1q21.1-1q44, 2q23.1-2q35, 7p22.3-7p11.1, 7q11.1-7q36.3 and 8q23.2-8q24.3.

**Table 2:** **CNA pattern of genomic areas exclusively deleted or amplified**

	Mean % of copy	Mean % of copy
	loss	gain
*Exclusively deleted cytobands*		
**1p36.33-1p35.1**	67.5%	1.0%
**3p26.3-3p14.25**	57.7%	3.4%
**14q24.1-14q32.33**	52.6%	0.0%
*Exclusively amplified cytobands*		
**1p11.2-1p41.1**	4.8%	17.9%
**1q21.1-1q44**	7.5%	29.5%
**2q23.1-2q35**	7.8%	20.2%
**7p22.3-7p11.1**	11.0%	24.7%
**7q11.1-7q36.3**	9.6%	22.6%
**8q23.2-8q24.3**	6.8%	22.6%

The average percentages for copy loss and copy gain are display for each genomic area.

The highest *exclusively* deleted cytobands were located in the terminal region of the short arm of chromosome 1 (1p36.33-1p35.1). This genomic region is deleted in numerous carcinomas but the impressive high rate of deletion in our series (range: 57.1-71.4%) may raise the issue of specificity for our calling procedure. However, the absence of CNAs detected in five cases having no or very few tumor cells as well as the absence of CNAs detected in normal samples analyzed in the same batch (data not shown) support the good specificity of our method. Sia *et al.* [19] reported a lower rate of copy loss (16%) but this result refers to the deletion of the whole short arm of chromosome 1. In their supplemental data, the high rate of the 1p36.32 to 1p35.2 focal region is concordant with our findings. The loss of 1pter region is not specific of non-fluke ICC since it has also been reported as the most frequent genomic aberration in fluke-associated ICC [43]. Four candidate tumor suppressor genes RUNX3, ARID1A, ERRFI1 and mTOR, all located on this exclusively deleted area, are of interest.

RUNX3 encodes a member of the runt domain-containing family of transcription factors and is considered as a tumor suppressor gene. Dachrut *et al.* [43] showed an inhibition through hypermethylation, associated with a decreased expression of the protein, suggesting the role of this protein in fluke associated carcinogenesis. ARID1A has emerged as a tumor suppressor gene, mutated in a broad spectrum of cancers, including ovarian clear cell, endometrioid, gastric and colon carcinomas. ARID1A gene plays the role of a gatekeeper (regulating cell cycle progression), but also of a caretaker (preventing genomic instability) [44]. ARID1A mutations result in loss of ARID1A-protein expression. It frequently co-occurs with PI3K/AKT-pathway activation and is associated with mismatch repair deficiency in colon cancer [45]. In a recent study, it has been shown that ARID1A-deficient cancer cells require the PI3K/AKT pathway and have an increased sensitivity to treatment targeting this pathway [46]. ARID1A is mutated in more than 10% of cholangiocarcinomas and is associated with copy loss of the genomic area [28]. ERRFI1 gene acts as a negative regulator for several EGFR family members, including EGFR and ERBB2 through direct interaction with the kinase domains of these proteins. Downregulation of ERRFI1 has been demonstrated in breast cancer and glioblastoma and more recently in a single case of cholangiocarcinoma [21]. Interestingly, the authors have shown that this patient underwent a rapid regression when treated with an EGFR inhibitor. It is worth noting that deletion of the 1p genomic area leads to a copy loss of the mTOR gene (1p36.22), encoding for a serine/threonine protein kinase which acts as a major downstream effector of the PI3K/AKT pathway.

The second *exclusively* deleted genomic area was 3p26.3-3p14.25. Two candidate genes (BAP1 and PBRM1) were located on this region. BAP1 (BRCA1-associated deubiquitylase) has been recently identified as a tumor suppressor gene, which promotes repair of DNA double-strand breaks, enhancing cell survival after DNA damage. BAP1 is mutated in several cancer types, including non-fluke ICC [28]. PBRM1 is involved in transcriptional activation and repression by chromatin remodeling and mutations have been found in ICC [29].

Surprisingly, the *exclusively* deleted 14q24.1-14q32.33 area harbors the AKT1 gene which is a serine/threonine kinase and plays a key role in cell deregulation as a downstream mediator of the KRAS/PI3K pathway. Our results are concordant with those from Sia *et al.* series [19], showing copy loss of 14q in 36% of the cases. It is worth noting that, in a lung cancer mice model, it has been shown that AKT1 deletion may prevent tumorigenesis by mutant KRAS [47].

The highest *exclusively* amplified genomic was 1q21.1-1q44. Gain of chromosome 1q copy is one of the most frequently detected alterations in hepatocellular carcinoma and has also been reported in cholangiocarcinomas. Some potential target genes (e.g. CHD1L) located on 1q21 have been recently reported [48].

The *exclusively* amplified genomic area 2q23.1-2q35 harbors IDH1 gene (Isocitrate Dehydrogenase 1). Gain of function mutations of the IDH1, leading to DNA methylation perturbation, have been reported in non-fluke ICC [21,28,29]. Our results raise the question of the potential role of amplification in IDH1 activation.

The whole chromosome 7 was amplified in eight cases which suggests a mechanism of aneuploidy. The chromosome 7 harbors numerous oncogenes, including: EGFR, MET, BRAF as well as the newly identified candidate KMT2C (MLL3). Mutating activation of EGFR [49], BRAF [11] as well as overexpression of MET [50] have been described in biliary carcinogenesis. It is interesting to note that the KMT2C (MLL3) gene is considered as a tumor supprossor gene and inactivating mutations have been reported in fluke-associated cholangiocarcinomas [28]. In our series, only five cases showed a deletion of the 7q36.1 cytoband harboring the KMT2C (MLL3) gene. The relationship between KMT2C (MLL3) mutation and copy number remains to be investigated.

The *exclusively* amplified cytoband 8q24.21 harbors MYC gene. It encodes for a nuclear protein which functions as a transcription factor that activates multiples genes and pathways. MYC has been shown to be activated by amplification in multiples carcinomas.

**Figure 4:**
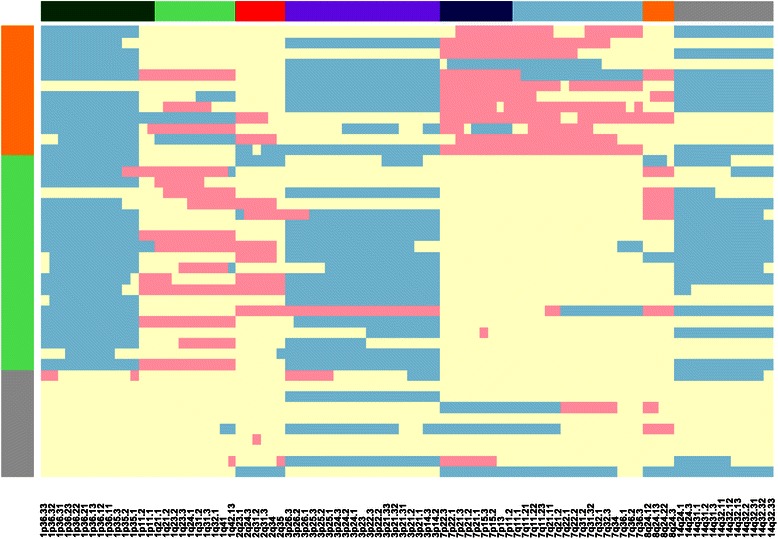
**Heatmap of the tumor samples for exclusively deleted and amplified cytobands.** The color coding for the matrix data is: copy loss (light blue), copy neutral (light yellow) and copy gain (pink). Three tumor clusters are indicated on the left side in orange (first class), light green (second class) and gray (third class). Cytoband clusters are shown on the top: dark green (1p), light green (1q), red (2q), purple (3p), blue (dark blue for 7p, light blue for 7q), orange (8q) and gray (14q).

We also analyzed how the tumor samples could be classified based on the minimal subset of *exclusively* deleted and amplified cytobands. The aim of this molecular classification was to identify tumor clusters defined by the smallest subset of *exclusively* deleted and amplified cytobands. The best model (according to the BIC criterion) corresponded to the one that led to three tumor clusters. Figure 4 shows a heatmap of the tumor samples for the *exclusively* deleted/amplified cytobands. Ten tumors are classified in the first cluster (orange in Figure 4) characterized by copy loss of 1p and copy gain of the short arm of chromosome 7. Twenty tumors are classified in the second cluster (ligth green in Figure 4) characterized by 1p and 3p copy losses and no 7p copy gain. Twelve tumors are classified in the third cluster (gray in Figure 4) characterized by no or very few alterations.

It is worth noting that all the cases of the first cluster, having copy gains of chromosome 7 showed also 1p copy loss. We may hypothesize that this tumor cluster is characterized by an activation of Growth Factor Receptors EGFR and MET (by chromosome 7 copy gain), amplified by the silencing of ERRFI1 (through 1p copy loss), a powerful negative regulator of several EGFR family members.

### Association between CNAs and pathological factors and RFS analysis

For each the *exclusively* deleted and amplified cytobands, we analysed the relationships between CNAs and the main pathological factors : size of the largest tumor, tumor number (single *vs* multiple), macrovascular invasion, histological vascular invasion and intraneural invasion, pathological stage and primary chemotherapy. After adjustment for multiplicity testing, none of these factors were associated with the distribution of the CNAs. Moreover, none of these clinico-pathological factors were associated with the total number of altered cytobands or the three tumor clusters.

**Figure 5:**
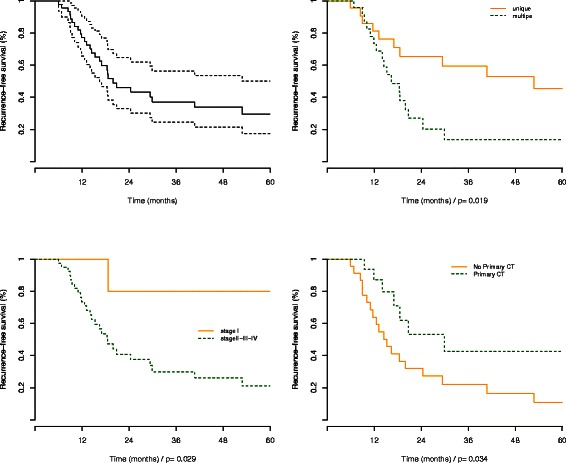
**Recurrence free survival (RFS) analyses for the entire series of patients (upper left) and according the main clinico-pathological factors: multiple versus unique tumors (upper right), pTNM stage (lower left) and primary chemotherapy (lower right).**

From univariate survival analysis, we found no relationship between time to recurrence and age (p=0.22) gender (p=0.14), histological differentiation (p=0.94), vascular (p=0.58), intraneural invasion (p=0.87), macrovascular invasion (p=0.57), resection margins (p=0.61). In contrast, multiple tumors (p=0.02), size of the largest tumor (p=0.04), pTNM 7^*th*^ edition (I vs. II-III-IV, p=0.03), were significantly associated with shorter time recurrence (Figure 5). In the group of advanced ICC (stage II-III-IV), primary chemotherapy was associated with a lower rate of recurrence (p=0.03).

We found no relationship between the total number of altered cytobands and the time to recurrence. We found no relationship between the three tumor clusters and the time to recurrence. We also found no relationship between time to recurrence and the 11q13.2 copy gain (p=0.37) or 14q22.1 copy loss (p=0.18) reported by Sia *et al.* [19].

In this work, we have selected a set of intrahepatic cholangiocarcinomas, from frozen material. This selection leaded to a relatively small sample size, which limits the statistical power of our analyses. This limitation, inherent to the rarity of this tumor, advocates for multi-institutional studies.

## Conclusion

This work describes the chromosomal CNA patterns of a series of ICC. We observed numerous copy number alterations in most of the samples with a high rate of exclusive deletion encompassing 1p, 3p and 14q. We also observed recurrent amplification for 1q, 7p, 7q and 8q. Some of these CNAs harbor candidate target genes. The co-occurence of copy gain of chromosome 7 and copy loss of 1p in a subset of tumor samples suggests an activation of EGFR receptor family together with a downregulation of the PI3K/AKT/mTOR pathway, raising the question of a potential sensibility of this subgroup to EGFR inhibitors or to therapies that target more specifically the RAS/MAPK signaling pathway. We do not find any relationship between CNAS and the clinico-pathological factors or the recurrence-free survival.
